# Analysis of the Relationship Between Motor Imagery and Age-Related Fatigue for CNN Classification of the EEG Data

**DOI:** 10.3389/fnagi.2022.909571

**Published:** 2022-07-14

**Authors:** Xiangyun Li, Peng Chen, Xi Yu, Ning Jiang

**Affiliations:** ^1^West China Biomedical Big Data Center, West China Hospital, Sichuan University, Chengdu, China; ^2^Med-X Center for Manufacturing, West China Hospital, Sichuan University, Chengdu, China; ^3^School of Mechanical Engineering, Southwest Jiaotong University, Chengdu, China; ^4^Department of Orthopedic Surgery and Orthopedic Research Institute, West China Hospital, Sichuan University, Chengdu, China; ^5^Rehabilitation Medicine Center, West China Hospital, Sichuan University, Chengdu, China; ^6^National Clinical Research Center for Geriatrics, West China Hospital, Sichuan University, Chengdu, China

**Keywords:** aging, brain–computer interfaces, motor imagery, fatigue, CNN

## Abstract

**Background:**

The aging of the world population poses a major health challenge, and brain–computer interface (BCI) technology has the potential to provide assistance and rehabilitation for the elderly.

**Objectives:**

This study aimed to investigate the electroencephalogram (EEG) characteristics during motor imagery by comparing young and elderly, and study Convolutional Neural Networks (CNNs) classification for the elderly population in terms of fatigue analysis in both frontal and parietal regions.

**Methods:**

A total of 20 healthy individuals participated in the study, including 10 young and 10 older adults. All participants completed the left- and right-hand motor imagery experiment. The energy changes in the motor imagery process were analyzed using time–frequency graphs and quantified event-related desynchronization (ERD) values. The fatigue level of the motor imagery was assessed by two indicators: (θ + α)/β and θ/β, and fatigue-sensitive channels were distinguished from the parietal region of the brain. Then, rhythm entropy was introduced to analyze the complexity of the cognitive activity. The phase-lock values related to the parietal and frontal lobes were calculated, and their temporal synchronization was discussed. Finally, the motor imagery EEG data was classified by CNNs, and the accuracy was discussed based on the analysis results.

**Result:**

For the young and elderly, ERD was observed in C3 and C4 channels, and their fatigue-sensitive channels in the parietal region were slightly different. During the experiment, the rhythm entropy of the frontal lobe showed a decreasing trend with time for most of the young subjects, while there was an increasing trend for most of the older ones. Using the CNN classification method, the elderly achieved around 70% of the average classification accuracy, which is almost the same for the young adults.

**Conclusion:**

Compared with the young adults, the elderly are less affected by the level of cognitive fatigue during motor imagery, but the classification accuracy of motor imagery data in the elderly may be slightly lower than that in young persons. At the same time, the deep learning method also provides a potentially feasible option for the application of motor-imagery BCI (MI-BCI) in the elderly by considering the ERD and fatigue phenomenon together.

## Introduction

Population aging is one of the severe challenges faced by all countries in the world nowadays and in the next few decades. According to the report of the Global Health and Aging published by WHO, by the middle of this century, the proportion of people over 65 years will increase from 11 to 22%, and the 85 years-and-over population is projected to increase by 351% between 2010 and 2050 (Mary et al., 2007). It also indicates that there will be a larger number of older people aged 60 years or over than adolescents aged 10–24 years by the middle of this century (Rudnicka et al., 2020). With the increase of age, the elderly generally have a decline in self-care ability and suffer from the risks of various diseases, which affect both their physical and mental health, leading to the degradation of the life quality in this group. Therefore, promoting the health level of the elderly is a critical factor in solving the aging problem.

As part of the aging process, a great number of changes occur in the central nervous system (CNS), and the brain function of the elderly is inevitably changed with the increase of age. Aging may also cause chronic neurological diseases which affect the motor system (Nikhil et al., 2014), and even healthy aging is accompanied by a decline in cognitive function (Gard et al., 2014). These kinds of cognitive or motor problems are reflected in the electrical activity of the brain and could be studied from electroencephalogram (EEG) signals. EEG analysis reveals the features of brain activities during the motor and cognitive tasks by various signal processing approaches (Pavlov et al., 2020). Many studies use EEG to explain the changes in the CNS due to the appearance of some aging-related diseases (Paiva et al., 2012). Brain–computer interface (BCI) establishes the direct interaction path between the brain and the external world by decoding the information from the brain during the mental tasks (Wolpaw et al., 2000). To help the elderly maintain a healthy, good quality of life, and sense of wellbeing, a number of BCI applications have been developed in recent years (Belkacem et al., 2020).

EEG could provide a non-invasive way to BCI with the characteristics of simple structure, high safety, and good real-time performance (Castermans et al., 2013). With the features extracted from the EEG signals, the classification based on the machine learning algorithms can convert them into the control commands for assistive or rehabilitation devices (Li et al., 2016). Several common paradigms are proposed to make the brain generate proper EEG signals. Generally, the EEG could be categorized as self-paced or non-self-paced ones. The self-paced ones only need the inner brain activities of the users (Müller-Putz et al., 2016), so these users can make their own decisions for control aims. In self-paced EEG, motor imagery (MI) is a dynamic cognitive process during which the movement is mentally simulated without actually being executed (Jeannerod, 2011). In the cerebral cortex, MI and motor execution of the same action have similar activity patterns. The goal of motor-imagery BCIs (MI-BCIs) is to control an external object by inducing and modulating the brainwaves of interest during the training sessions so that the BCI system can determine the user's intention in real-time in testing sessions (Jiang et al., 2022). Several studies have explored the influences of aging on different aspects of MI, such as vividness (Malouin et al., 2010), working memory (Schotta, 2012), and the temporal performance of the MI (Personnier et al., 2010). However, these studies are based on scales and statistics analysis, and the EEG indicators have not been introduced to evaluate the difference in MI ability across different age groups.

EEG sensorimotor rhythm changes from the resting state to the MI or motor execution state. This phenomenon reflects a decrease in the power of EEG over the primary sensorimotor area in the alpha (7–4 Hz) and beta (15–35 Hz) bands indicating underlying cortical cells to be desynchronized, which is named as event-related desynchronization (ERD) (Hisato et al., 2018). A comparative study of the ERD of EEG signals during MI tasks in different age groups would be helpful to understand the changes in cerebral cortex activity and the influence of aging on improving the MI training in neurological rehabilitation and BCI system design for the elderly.

In the MI-BCI, it is significant to make a cognitive effort to focus on MI tasks, but the EEG signal quality heavily depends on the mental state, level of attention, and fatigue of BCI users. Loss of attention due to mental fatigue significantly decreases signal characteristics, and thus reduces the performance of BCI systems (Cao et al., 2014). Some studies (Talukdar et al., 2018) also have confirmed that long-term MI can cause mental fatigue, and the level of mental fatigue corresponds to the EEG data separability of MI, which relates to the further application of MI-BCI. However, most of the current studies were only conducted on young adults. Aging increases the complexity of human brains (Anokhin et al., 1996), and this effect is demonstrated in the frontal inferior and sensorimotor areas (Scheel et al., 2018). In addition, entropy-based tools are widely used to quantify complexity, and approaches related to time–frequency analysis in separate frequency bands provide a clear physiological interpretation of the changes in EEG signals (Pavlov et al., 2020). Thus, it is necessary to investigate the fatigue caused by MI from the aspects of EEG power on both frontal inferior and sensorimotor regions.

With the different fatigue characteristics of EEG in the young and elderly population, a suitable MI data classification method is crucial for generating the control commands for the assistive and rehabilitation device application in aging groups. In the MI data classification stage, the extracted features are interpreted as the BCI user intentions, and machine learning is the most frequently used classification method. Deep learning belongs to representation learning, which aims to better represent input data using multiple layers of processing blocks such as neural networks (e.g., CNN and RNN). CNN (Lecun et al., 1989), proposed for the first time in the 1980s, was used to classify handwritten digits. CNN is of vital importance for EEG decoding using deep learning methods. To introduce CNN algorithms to EEG data classification, two measures are generally taken. First, in the aspect of the good performance of CNN for image classification, many researchers firstly convert the EEG signal into image information and then input it into CNN for classification. It uses the time–frequency maps of C3, Cz, and C4 channels at 6~13 Hz and 17~30 Hz for splicing as the feature of EEG data input to the convolutional neural network (CNN) for classification (Tabar and Halici, 2017). Another idea is to utilize the “end-to-end” characteristics of deep learning by importing the preprocessed EEG data as the input of the neural network and directly extracting the deep abstract features in the EEG data through the neural network. EEGNet (Lawhern et al., 2018) builds a deep learning model based on a CNN to directly classify EEG data samples, and the core idea is to convolve the channel dimension and the time dimension, respectively, and directly extract and classify features from these two dimensions. In the previous studies, the classification of EEG signals by CNN is usually applied to younger subjects, while the comparative study of young adults and the elderly has not been thoroughly carried out. If this effectiveness of the tool is also verified on MI data from older groups, it will promote the development of rehabilitation and assistive BCI more suitable for the elderly population and facilitate their everyday lives.

This study investigates the ERD of EEG signals in MI tasks in different age groups to clarify the differences in MI ability in both groups. The changes in fatigue level during MI will be studied. Fatigue analysis includes three aspects: the first is to distinguish fatigue-sensitive channels in the parietal region; another is to calculate rhythm entropy (RE) in the frontal region; the third is to analyze whether the fatigue in the parietal region and the EEG complexity in the frontal lobe are synchronized through phase-locked values (PLVs). Based on such analysis, it investigates the differences in the classification performance of deep learning methods (CNN) on young and the elderly, which will provide a reference for designing BCIs for rehabilitation and daily living aids used by the elderly.

## Methods

### Participants

Ten young adults (S1–S10, 8 males and 2 females, mean age 23) and 10 older adults (T1–T10, 3 males and 7 females, mean age 66) participated in this study. All participants were right-handed and had a normal or corrected vision, without known neurological or psychological disorders, use of psychiatric drugs, or any drugs affecting the CNS. The participants were asked to have a good sleep before the experiment. Each participant was informed about the experimental protocol and included after receiving verbal consent for the experimental trials. The protocol studied was approved by the local ethical committee and performed in accordance with the Declaration of Helsinki.

### EEG Recording

EEG data were acquired with a 64-channel EEG cap (Waveguard Original, ANT Neuro b.v., Enschede, Netherlands) and a mobile EEG amplifier (eego sports, ANT Neuro b.v., Enschede, Netherlands) at a sampling rate of 1,024 Hz. The 64-electrode configuration was set according to the international 10–20 system. The reference and ground electrodes were placed on CPz and AFz, respectively. Most electrodes (about 65%) had impedances <5 KΩ.

### Experiment Setup and Paradigm

**Figure 1:**
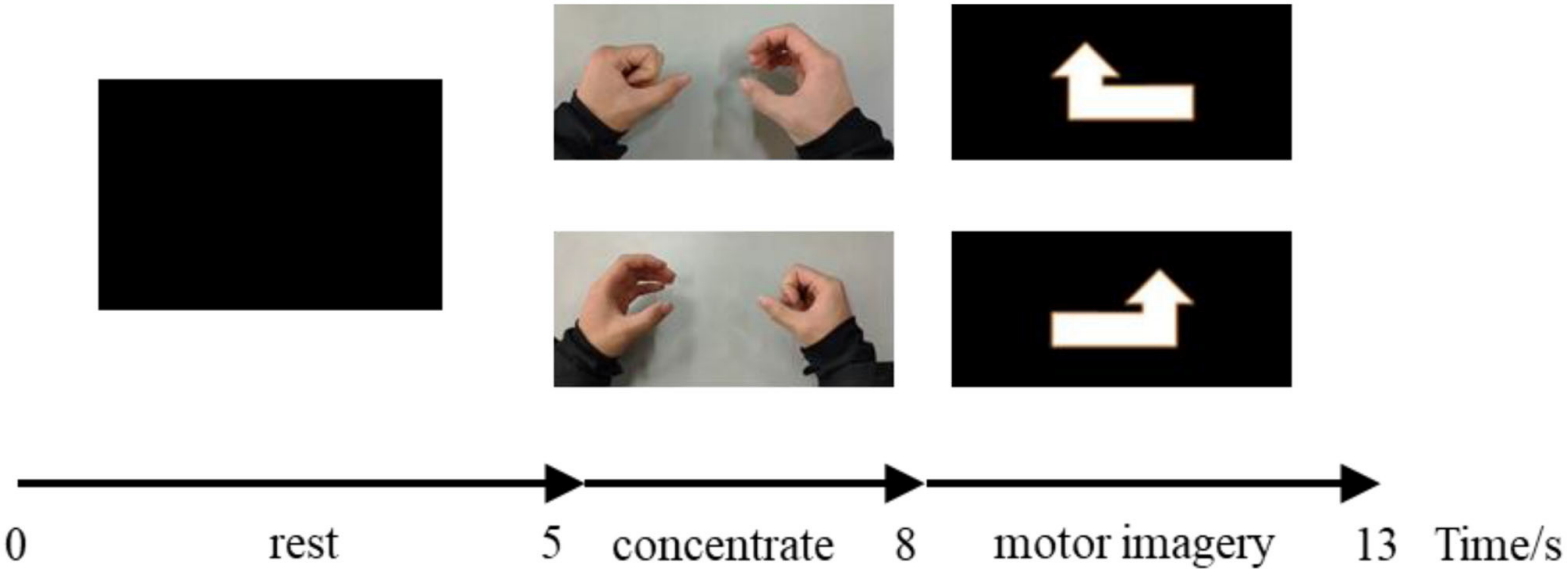
Experimental protocol for a single trial.

Participants are asked to sit in a comfortable chair and look at the computer screen, which displays the experimental paradigm. Participants are asked to avoid blinking and body movements during EEG recording. The two different MI tasks (left-hand movement vs. right-hand movement) are performed by the participants. The experimental session consists of four runs of continuous EEG signal recording. Each participant performs 40 trials in each run, with a total of 160 trials. During each trial, the computer screen first displays a black screen for 3 s, then a video of the left or right-hand movement of the fist for 3 s, and finally a 5-s up-left or up-right arrow together with 30 s rest for every 10 trials. Participants keep relaxed when the screen is black, concentrate when the video is playing, and begin to imagine the corresponding movement when the arrow appears. The sequence of events in the trial is illustrated in Figure 1, and a 5-min rest is given between runs. It should be noted that S8 collected two more runs for some reasons, reaching 240 samples.

### EEG Data Pre-processing

The EEG signals were processed using the EEGLAB toolbox. A 50 Hz industrial frequency interference has been removed and the band-pass was filtered from 1 to 35 Hz using a finite impulse response (FIR) filter. The filtered data is resampled to 512 Hz. Then, the resample data was segmented to extract epochs and removed baseline. This resulted in a total of 160 epochs for each participant, except for S8. All artifacts were removed in each epoch (such as eyeblinks and body movement) by independent component analysis (ICA). The attributes of components were observed by the ICLabel function, and the components which belong to EOG, EMG, noises, and other artifacts were discarded, and only those with more than 60% probability of being estimated as EEG were retained. The run with more artifacts in the four runs of experiments was eliminated in young adults when ERD and fatigue were analyzed, but all runs were retained in older adults.

### Event-Related Desynchronization/Synchronization (ERD/S)

To analyze the fatigue levels in the process of MI, it needs to verify the ERD/ERS phenomenon in the process of MI through time–frequency analysis and the results are used for time–frequency analysis to carry out the subsequent related calculation of fatigue. The time–frequency analysis implemented by Letswave7.ERD/ERS is calculated in a defined frequency band about a baseline reference interval. The baseline reference interval for the ERD/ERS calculation was taken from 0 to 1 s for each epoch. After performing the artifact removal mentioned above, wavelet transformation was applied to analyze the law of EEG signal energy changing with frequency and time. The single time–frequency map under the same task is superimposed and averaged to obtain the final time–frequency map.

### Fatigue Analysis in Motor Imagery

The α and β EEG signals increase and θ EEG signal decrease during fatigue, and the (θ+α)/β has the strongest positive correlation with fatigue (Jap et al., 2009). This paper fuses (θ+α)/β and θ/β into a comprehensive fatigue index to analyze and evaluate the fatigue level in real-time. The preprocessed EEG signal is decomposed and reconstructed by wavelet transform, and the EEG signal of different characteristic frequency bands (θ, α, and β frequency bands) is extracted. The EEG signal has strong individual differences, and the EEG channels sensitive to fatigue differ among subjects. It calculates the correlation between the fatigue index and the experimental time of each channel, selects the channel whose correlation value is larger than the threshold, and then sets it as the sensitive channel. According to experience, the threshold is set to 0.75 in this paper. Fatigue-sensitive channels in the parietal lobe are extracted, which are located in the center of the human brain that processes sensory. After that, the power spectral density of each sensitive channel is calculated separately, and the fatigue level is finally obtained.

Entropy is robust in assessing the regularity and predictability of complex systems and has been used to analyze EEG signals (Arunkumar et al., 2018). RE extracts each rhythm of the EEG signal and then calculates its entropy value. For the six channels (F3, F4, F5, F1, F2, and F6) located in the frontal cortex of each subject, their RE is calculated.

First, calculate the energy for each trial of different rhythmic cortical activity:


$$Power=\sum_{i=1}^{m}S{\left(x\right)}^{2}$$

where *S(x)* represents estimated cortical activity and *m* is the number of sample points.

Then, the energy normalized by the *j*th frequency band rhythm cortical activity is calculated, and the value is obtained by dividing the sum of the energies of the three frequency bands according to the following equation:


$$P_{j}=\frac{Power_{j}}{\sum_{j=1}^{k}Power_{j}}$$

where *Power*_*j*_ represents the energy of the *j*th frequency band rhythm cortical activity and *k* is the number of the frequency band.

The formula of RE calculation is:


$$En=-\sum_{j=1}^{k}P_{j}log_{2}P_{j}$$

To verify the selection of parietal lobe fatigue channels and the rationality of prefrontal lobe RE fatigue analysis, the synchronization of the channels was verified by calculating the PLVs of fatigue-sensitive channels and prefrontal lobe channels. For two univariate continuous-time signals, *x*(t) and *y*(t), the phase synchronization relationship can be expressed by the PLV.


$$\begin{array}{l} PLV=\left|{\langle e^{i\phi_{xy}(t)}\rangle }_{t}\right|=\sqrt{{\langle cos\phi_{xy}(t)\rangle }_{t}^{2}+{\langle sin\phi_{xy}(t)\rangle }_{t}^{2}}\\ \;\phi_{xy}\left(t\right)=\phi_{x}\left(t\right)-\phi_{y}(t) \end{array}$$

where, < > represents the average value of time and Φ_*x*_(*t*) and Φ_*y*_(*t*) represent the instantaneous phases *x*(*t*) and *y*(*t*), respectively. According to the above formula, the PLVs of any two time-signals *x*(t) and *y*(t) can be obtained. If PLV = 0, it means that *x*(t) and *y*(t) have no phase synchronization. If PLV = 1, then *x*(t) and *y*(t) synchronize in their phases. The range of PLVs is [0,1].

### Proposed CNN Architecture

**Figure 2:**
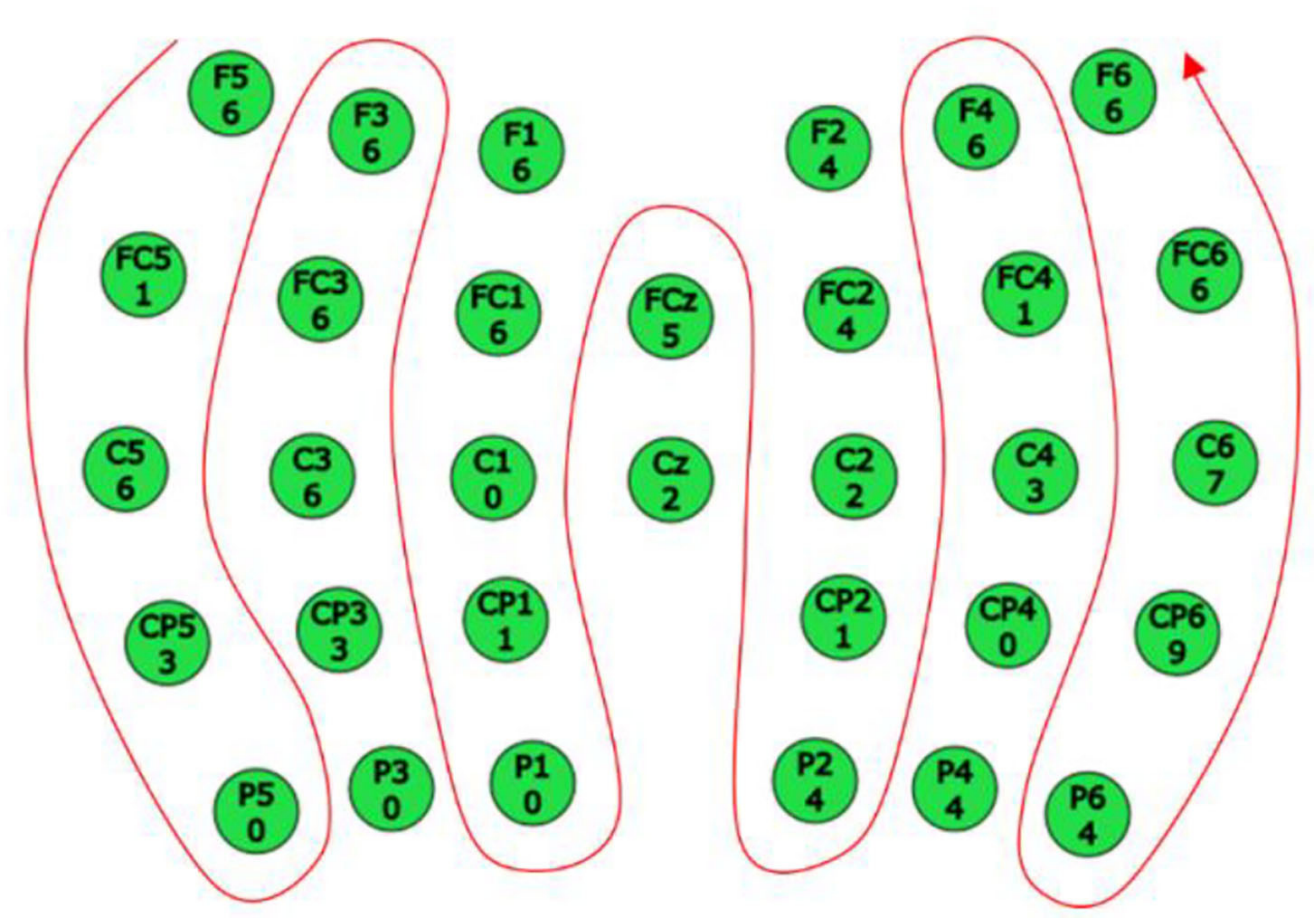
The rearranged order of the selected 32 channels.

**Figure 3:**
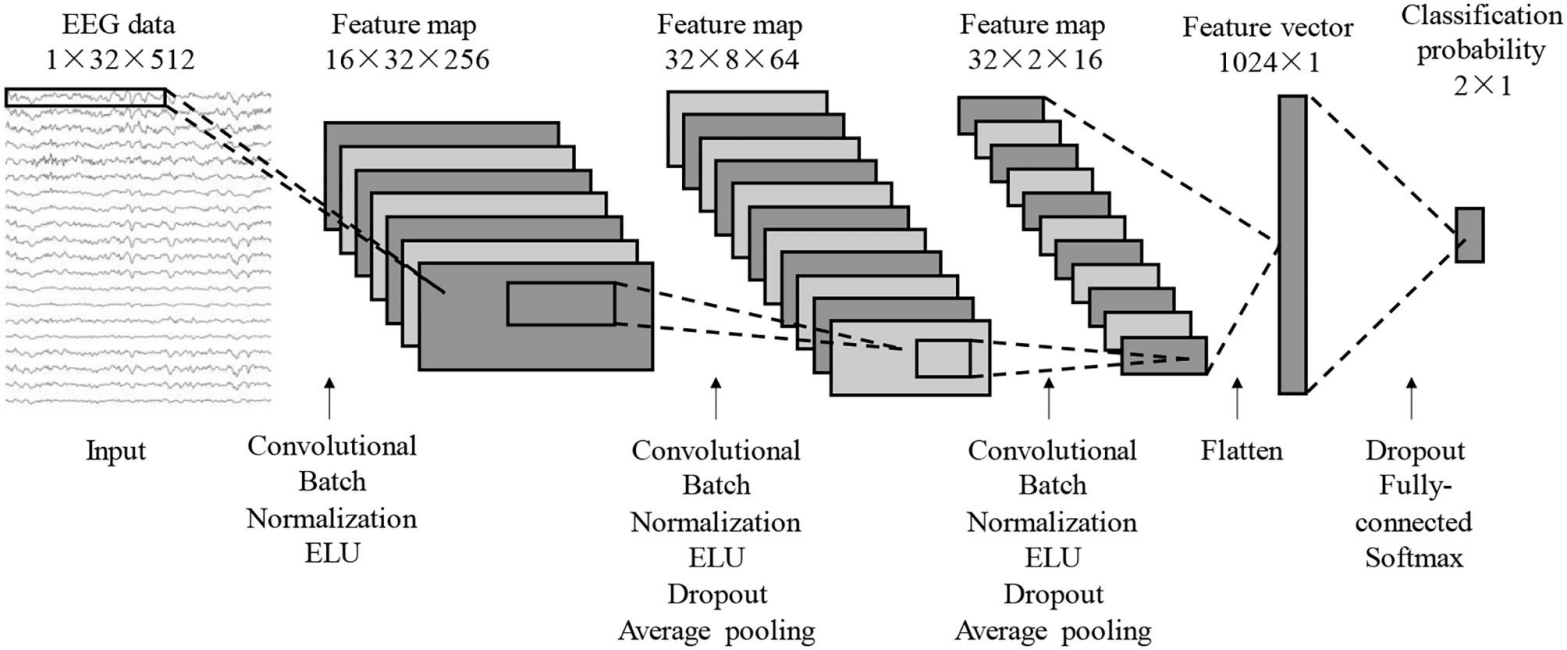
CNN structure diagram that fuses spatial information.

Referring to the network structure of EEGNet, combined with the MI characteristics of the young and elderly, a CNN algorithm that integrates spatial information to classify and identify MI-EEG signals is proposed. It is of great help to improve the classification accuracy and enhance the generalization ability of the CNN model by extracting time, frequency, and space information from the EEG signals (Yimin et al., 2020). To preserve the spatial information on the ipsilateral side and distinguish that on the opposite side, the rearranged order of the selected 32 channels, as shown in Figure 2, was as follows: F5, FC5, C5, CP5, P5, P3, CP3, C3, FC3, F3, F1, FC1, C1, CP1, P1, Cz, FCz, F2, FC2, C2, CP2, P2, P4, CP4, C4, FC4, F4, F6, FC6, C6, CP6, and P6. In addition, it normalized the samples as a whole. The standardization method was to subtract the mean value of all the data in the sample and divide it by the standard deviation. After normalization, all data points of each sample fit a distribution with mean 0 and standard deviation 1, which helped to speed up the rate of gradient descent in CNN. The MI-EEG classification problem in this paper belongs to a small sample set classification problem. To avoid the overfitting phenomenon caused by the overcomplicated model, the CNN structure designed in this paper only contains three convolutional layers, with two pooling layers and one fully connected layer, as shown in Figure 3. Based on the classic EEGNet, the structure modifies the size of the convolution kernel and the convolution stride.

As shown in Figure 3 and Table 1, the dimension of a single sample input to CNN is 1 × 32 × 512. The size of the convolution kernel of the first layer of convolution is 1 × 24, the stride of the convolution kernel is 1 × 2, and the mode of partial filling is adopted. The first layer of convolution is mainly to perform time-domain convolution of one-dimensional signals. Its function is to refine the characteristics of each channel in the time domain signal. The main purpose is to reduce the length of the signal in the time dimension. The number of feature map channels output by the first layer of convolution is 16. The second layer of convolution uses a 2 × 11 convolution kernel, and the convolution stride is 2 × 1. This convolution operation can integrate the information of adjacent channels while keeping the signals of the left and right brain channels from mixing. After the second layer of convolution, the pruning operation is performed at a ratio of 0.25. The pruning randomly makes some neurons inactive, thereby avoiding overfitting in the neural network. This is followed by the first pooling layer with a pooling kernel size of 2 × 4. The pooling layer can extract important information from the feature map computed by the convolutional layer. As the feature map is much longer than the width, a 2 × 4 pooling kernel is used. The third layer of convolution uses a 2 × 5 convolution kernel, and the stride of the convolution is still 2 × 1. After that, pruning is performed at a ratio of 0.25, and a 2 × 4 pooling operation is also performed. The padding parameter of all convolution operations is set to 0, i.e., no padding mode. In addition, batch normalization is performed after each convolution operation, and the activation is performed using the exponential linear unit (ELU) activation function. The batch normalization operation can speed up the learning of the neural network and reduce the dependence on the initial weights. The ELU activation function is an improved version of the ReLu activation function. Compared with the ReLu activation function, the average value of its output is close to 0, which will not add additional bias to the next layer of neurons, thereby speeding up the convergence of the model. In the negative case, it has soft saturation characteristics and is more robust to noise.

**Table 1:** Implementation details for proposed CNN architecture.

**Type**	**Maps**	**Size**	**Kernel size**	**Stride**	**Padding**	**Parameter**
Input	1	32 × 512	–	–	–	0
Convolutional 1	16	32 × 256	1 × 24 × 16	1 × 2	0 × 11	400
Batch normalization 1	16	32 × 256	–	–	–	0
Activation 1	16	32 × 256	–	–	–	0
Convolutional 2	32	16 × 256	2 × 11 × 32	2 × 1	0 × 5	11,296
Batch normalization 2	32	16 × 256	–	–	–	0
Activation 2	32	16 × 256	–	–	–	0
Average pooling 1	32	8 × 64	2 × 4	2 × 4	0	0
Spatial dropout 1	32	8 × 64	–	0.25	–	0
Convolutional 3	32	4 × 64	2 × 5 × 32	2 × 1	0 × 2	10,272
Batch normalization 3	32	4 × 64	–	–	–	0
Activation 3	32	4 × 64	–	–	–	0
Average pooling 2	32	2 × 16	2 × 4	2 × 4	0	0
Spatial dropout 2	32	2 × 16	–	0.25	–	0
Flatten	–	1,024	–	–	–	0
Fully-connected	–	2	–	–		2,050
Softmax	–	2	–	–		4
Total						24,022

## Results

### Time–Frequency Diagram of the ERD/ERS

**Figure 4:**
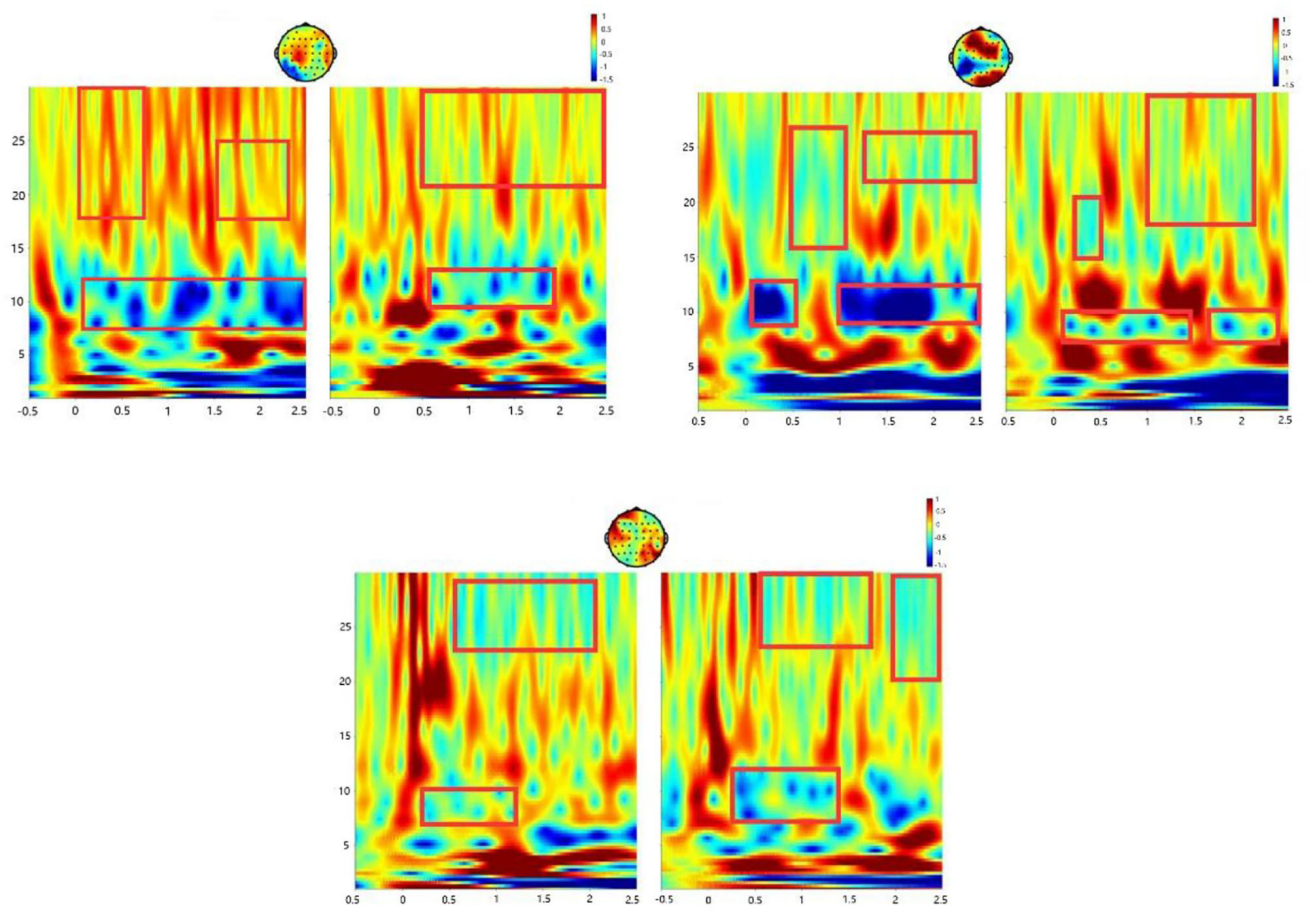
ERD/ERS time–frequency analysis of three groups in S1.

To analyze the fatigue of young and elderly subjects and find out the difference between them, it needs to prove that the subjects have obvious ERD/ERS phenomenon in the process of MI and then carry out fatigue calculation on this theoretical basis. For this purpose, the most representative C3 and C4 channels in the sensorimotor area of the cerebral cortex were selected for analysis, and the time–frequency diagram of the channels of the S1 subject is shown in Figure 4. The desynchronization at channels C3 and C4 is visually more pronounced in the alpha–beta (8–30 Hz) frequency band. The same subject had different levels of desynchronization in different periods and showed a trend of decreasing ERD significance with the increase of experimental time.

Table 2 gives the quantified average value of ERD of C3 and C4 channels within 0~1 s and 1~2.5 s in the alpha frequency band, and *t*-tests were performed, respectively. In both channels, the desynchronization phenomenon in the 1~2.5 s time period is more significant than that in the 0~1 s time period (C3: *P* < 0.05; C4: *P* < 0.05). It is regarded that 0~1 s is the preparation period of MI, and 1~2.5 s is the execution period of MI. Through the above process, other subjects were also verified, and the conclusion could prove that these subjects showed obvious ERD/ERS phenomenon in the MI process.

**Table 2:** *T*-test of ERD phenomena for C3 and C4.

	**Channel**	**Band**	**ERD quantitative values**	
			**0**~**1 s**	**1**~**2.5 s**
S1-1	C3	α (8~13Hz)	−0.45044	−0.94739
S1-2	C3	α (8~12Hz)	−0.31794	−1.0896
S1-3	C3	α (9~12Hz)	−0.56522	−1.6016
Significant	0.021975815			
S1-1	C4	α (8~12Hz)	0.026749	−0.51328
S1-2	C4	α (8~13Hz)	−0.047736	−0.34357
S1-3	C4	α (9~12Hz)	0.20375	−0.15104
Significant	0.036622313			

### Results of the Fatigue Analysis During Motor Imagery

**Figure 5:**
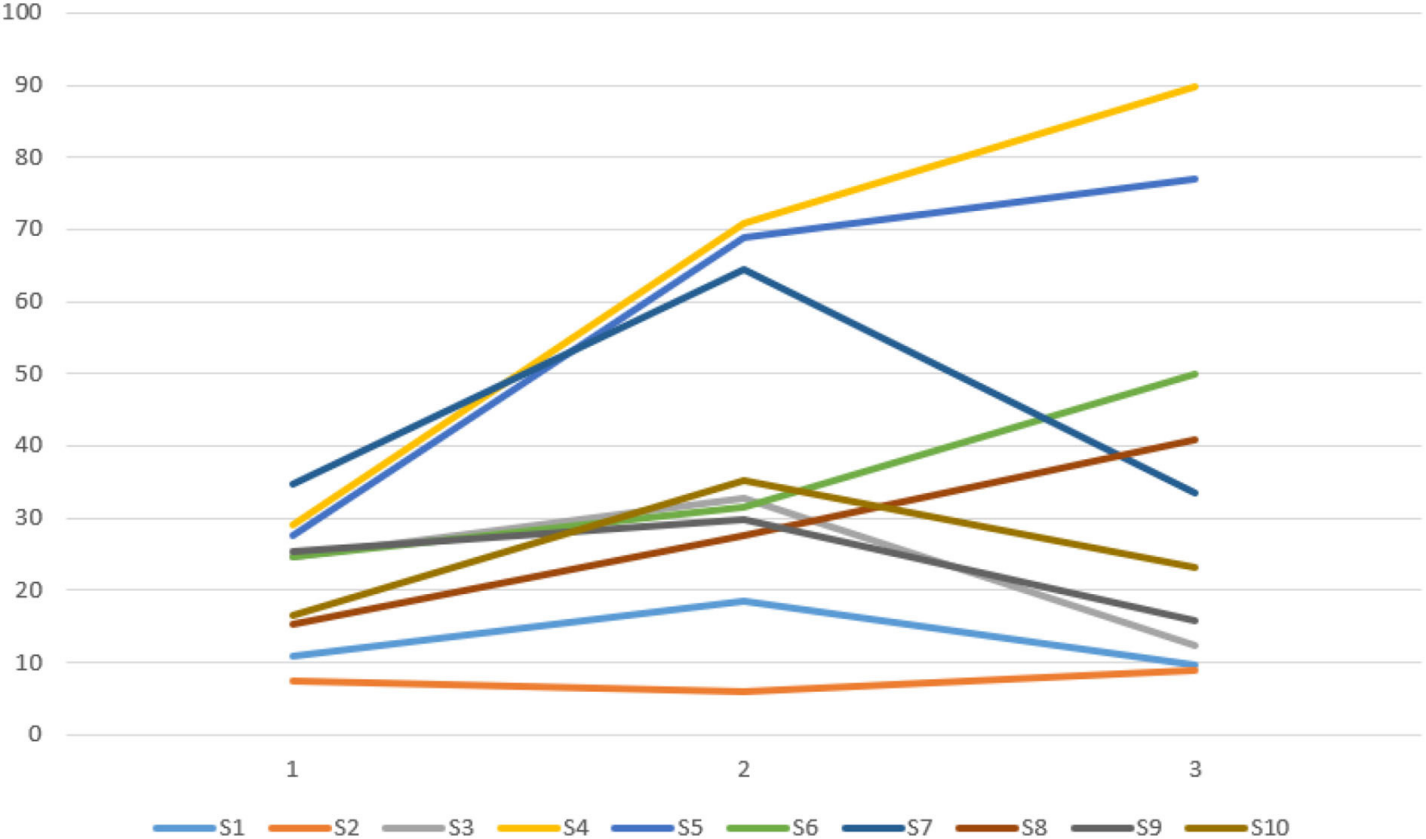
Relationship between fatigue changes over time in 10 young subjects.

**Figure 6:**
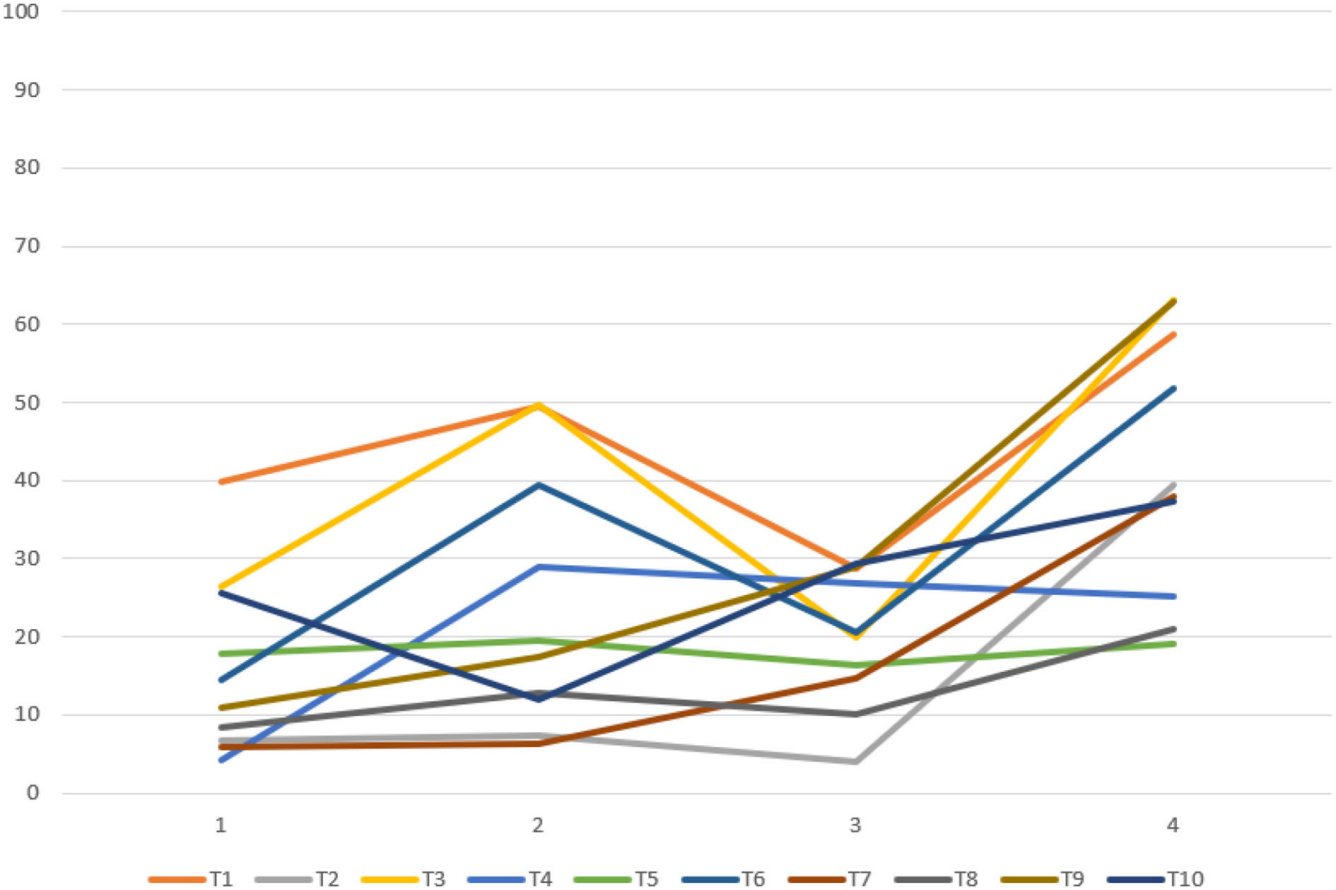
Relationship between fatigue changes over time in 10 elderly subjects.

Based on verifying the ERD/ERS phenomenon, it can conduct fatigue analysis on the parietal lobe channels of the subjects according to the fatigue calculation method mentioned above for selecting the fatigue-sensitive channels. The fatigue-sensitive channels of young and older participants in the parietal lobe areas are shown in Table 3. In this group of young participants, the P6 channels were more sensitive to fatigue, and the P5 channels were relatively less sensitive to fatigue. The P2 channels were more sensitive to fatigue in elderly subjects, and the P6 channels were relatively less sensitive to fatigue. The relationship between fatigue and test time is shown in Figures 5, 6. For most of the young subjects, the increase in the experimental time did not have a strong effect on the fatigue level, and the general trend was that the fatigue level increased with the duration of the experimental time. For most older subjects, the fatigue showed a general increasing trend with the experimental time. Moreover, the increase in experimental time had less effect on fatigue for them than for the young ones.

**Table 3:** The fatigue-sensitive channels of the young and elderly subjects on the parietal lobe.

	**Subject**	**Fatigue-sensitive**	**Subject**	**Fatigue-sensitive**
		**channels**		**channels**
The young subjects	S1	P1 P6	S6	P7 P3 P4 P2 P6
	S2	P4 P8 P2 P6	S7	P7 P3 P4 P1 P6
	S3	P3 P8 P1 P2 P6	S8	P7 P4 P8 P2 P6
	S4	P7 P3 P4 P1 P6	S9	P4 P8 P2 P6
	S5	P7 P4 P1 P8 P6	S10	P7 P3 P4 P8 P2
The elderly subjects	T1	P3 P4 P1 P2	T6	P3 P4 P8 P1
	T2	P4 P5 P1 P2 P6	T7	P7 P3 P8 P5 P1 P6
	T3	P7 P3 P5 P1 P2	T8	P7 P3 P4 P5 P1 P2
	T4	P7 P4 P8 P2 P6	T9	P7 P3 P4 P5 P2
	T5	P7 P4 P8 P5 P2 P6	T10	P4 P8 P1 P2

Fatigue is a set of events produced by labor or prolonged exercise and can influence the performance of most tasks, since it contributes to the reduction of perceptual, cognitive, and motor skills (Tello et al., 2014). The parietal lobes are responsible for spatial reasoning and motor information processing, while the frontal lobes are involved in planning and higher cognitive abilities (Rao, 2013). Entropy-based feature analysis can obtain the relationship between the complexity of the EEG signal and the fatigue state of the subjects (Hu and Min, 2018). Lehmann et al. (2022) observed that prefrontal activity was generally a more important predictor of balance performance in older adults than in younger ones. The above analysis of fatigue is only done in the parietal lobe area. Here, RE is used to detect fatigue changes in the frontal lobe area.

**Figure 7:**
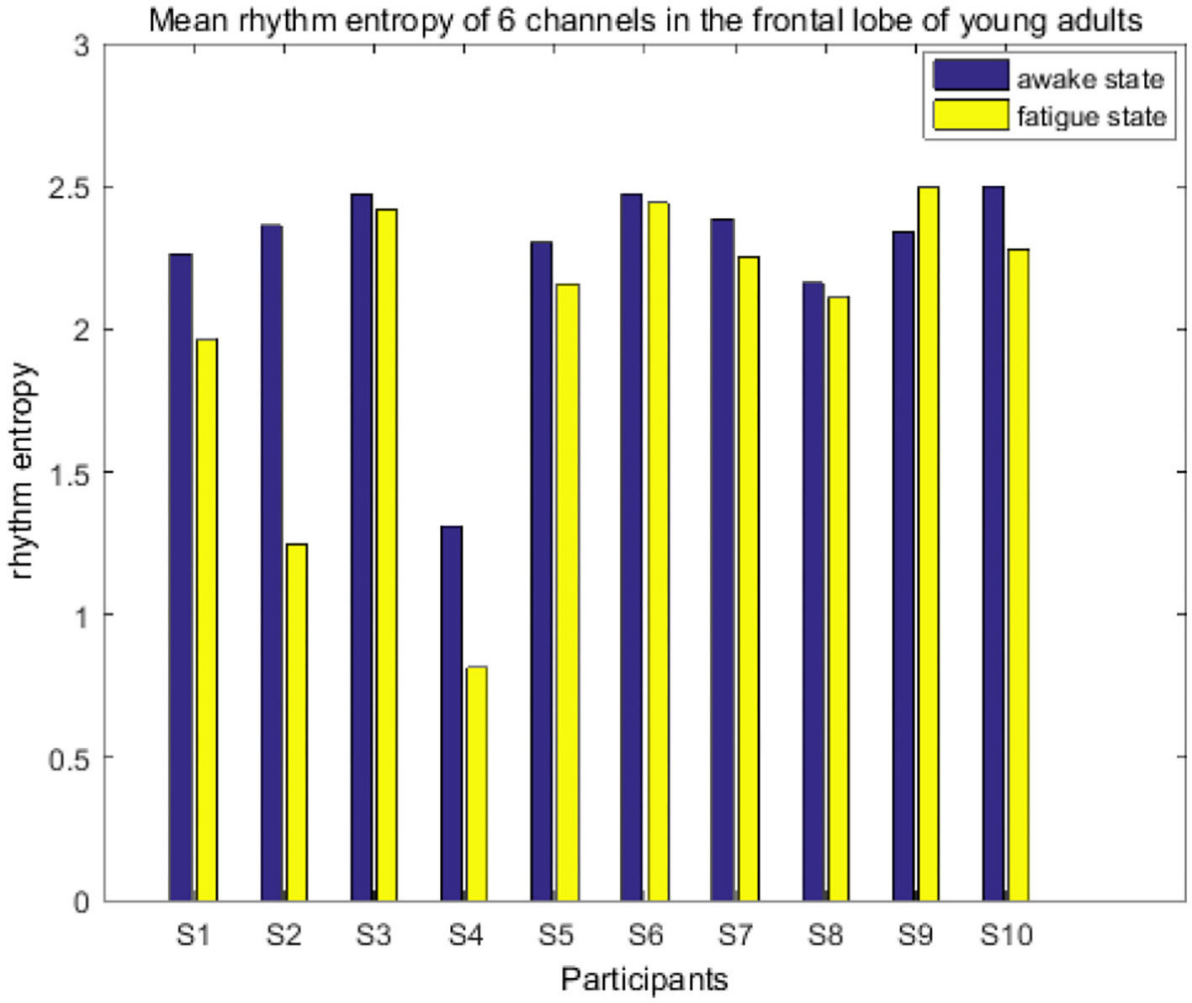
Mean rhythm entropy of six channels on the frontal lobe of young adults.

**Figure 8:**
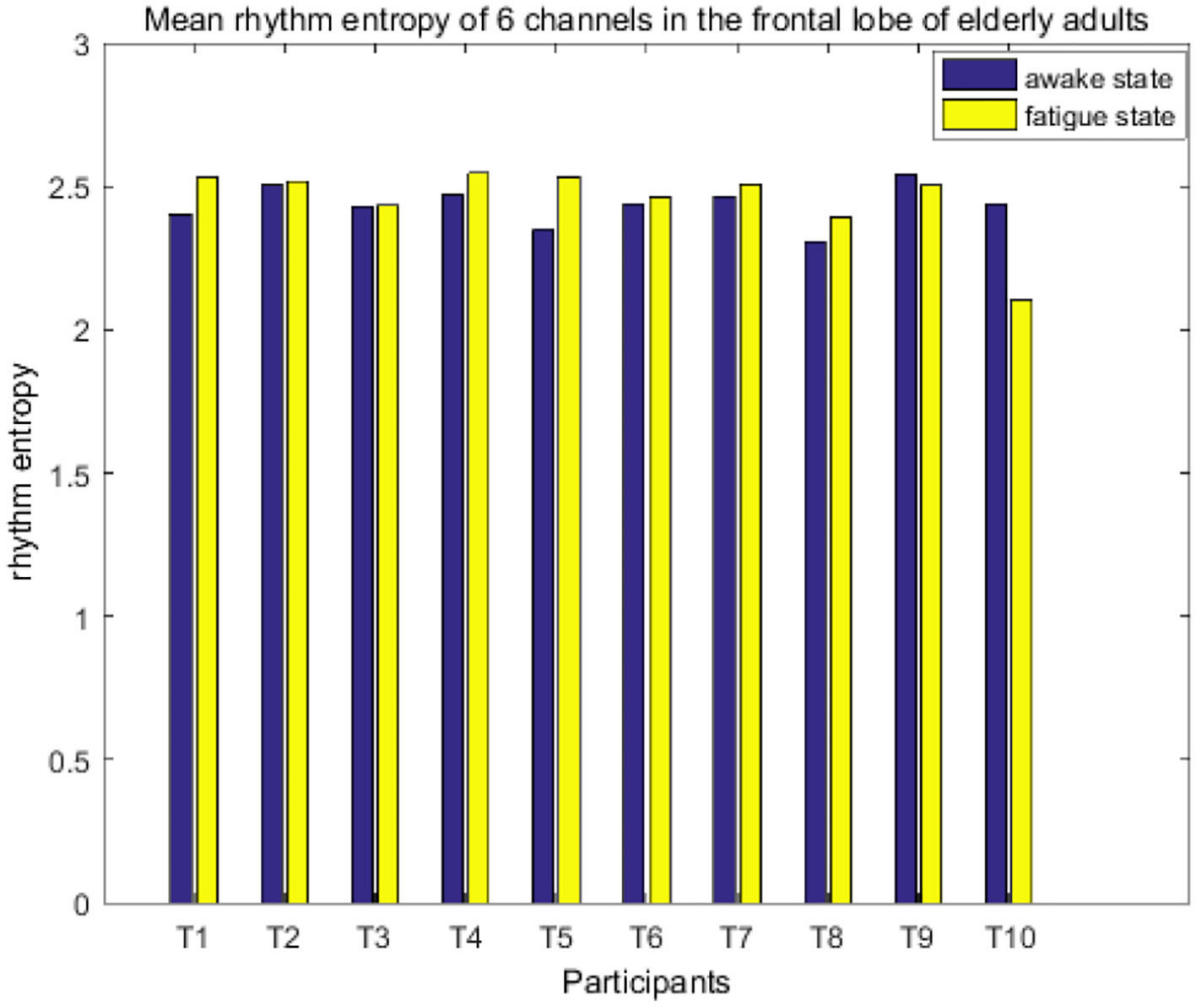
Mean rhythm entropy of six channels on the frontal lobe of elderly adults.

It treated the first 20 MI trials as awake state and the last 20 trials as fatigue state, and then calculated the average of the RE of the six channels of the frontal cortex (F3, F4, F5, F1, F2, and F6) in the awake state and the fatigue state of the 20 participants. As shown in Figures 7, 8, RE is roughly equal in young and elderly in the awake state, while such value in the fatigued state is lower than that in the awake state in the youngest subjects (9 participants) but higher in the most elderly ones (8 participants). The RE changes of subjects S9 and T9 differed from others in their own groups, with the changes not obvious between the two states, which may be due to individual differences, especially for subject T10. Human brain activity is complex in the awake state, so the RE is high. When the brain activity becomes orderly and complexity is reduced in the fatigued state, it results in a decrease in RE. From the RE variations, it can infer that for the young adults, the fatigue occurred accompanied by the execution of the MI, while older adults remain alert during the experiment in that they might require greater cognitive effort to complete MI tasks.

**Figure 9:**
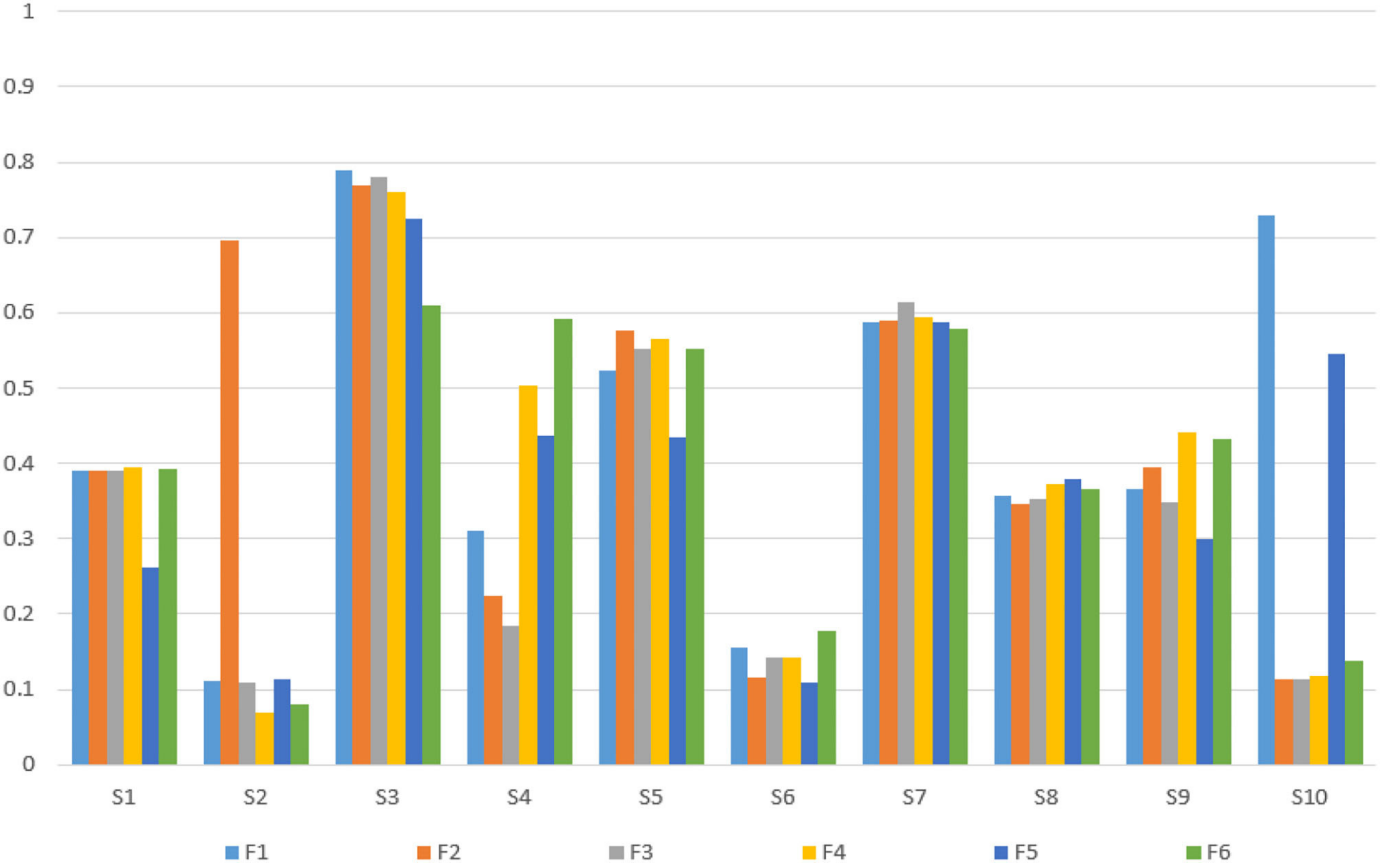
PLV of fatigue sensitive channel P6 to frontal channels of 10 young subjects.

**Figure 10:**
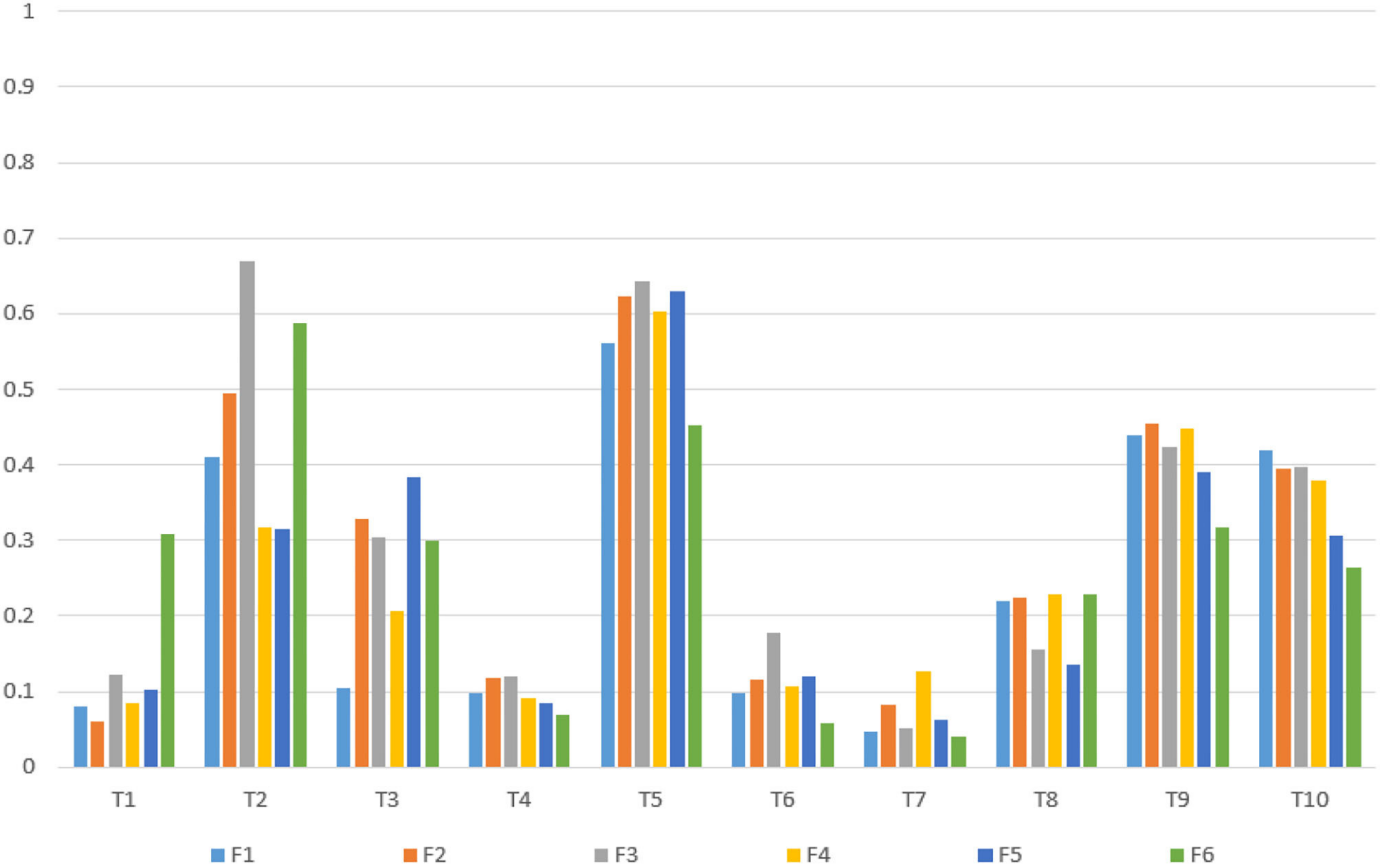
PLV of fatigue sensitive channel P2 to frontal channels of 10 elderly subjects.

Combining with the above conclusion, it selected the 10 groups of young subjects fatigue sensitive channels (P6) and 10 groups of elderly subjects fatigue sensitive channels (P2), and the PLV of P6 and P2 to 6 frontal channels (F3, F4, F5, F1, F2, and F6) were calculated, respectively. Figure 9 shows the calculation results of PLVs for 10 young subjects, and Figure 10 shows the results of PLVs for 10 older subjects. It can be found that there is a high synchronization between the fatigue-sensitive pathway and the frontal cortex pathway for both young and old subjects.

### Results of the Classification by CNN

**Figure 11:**
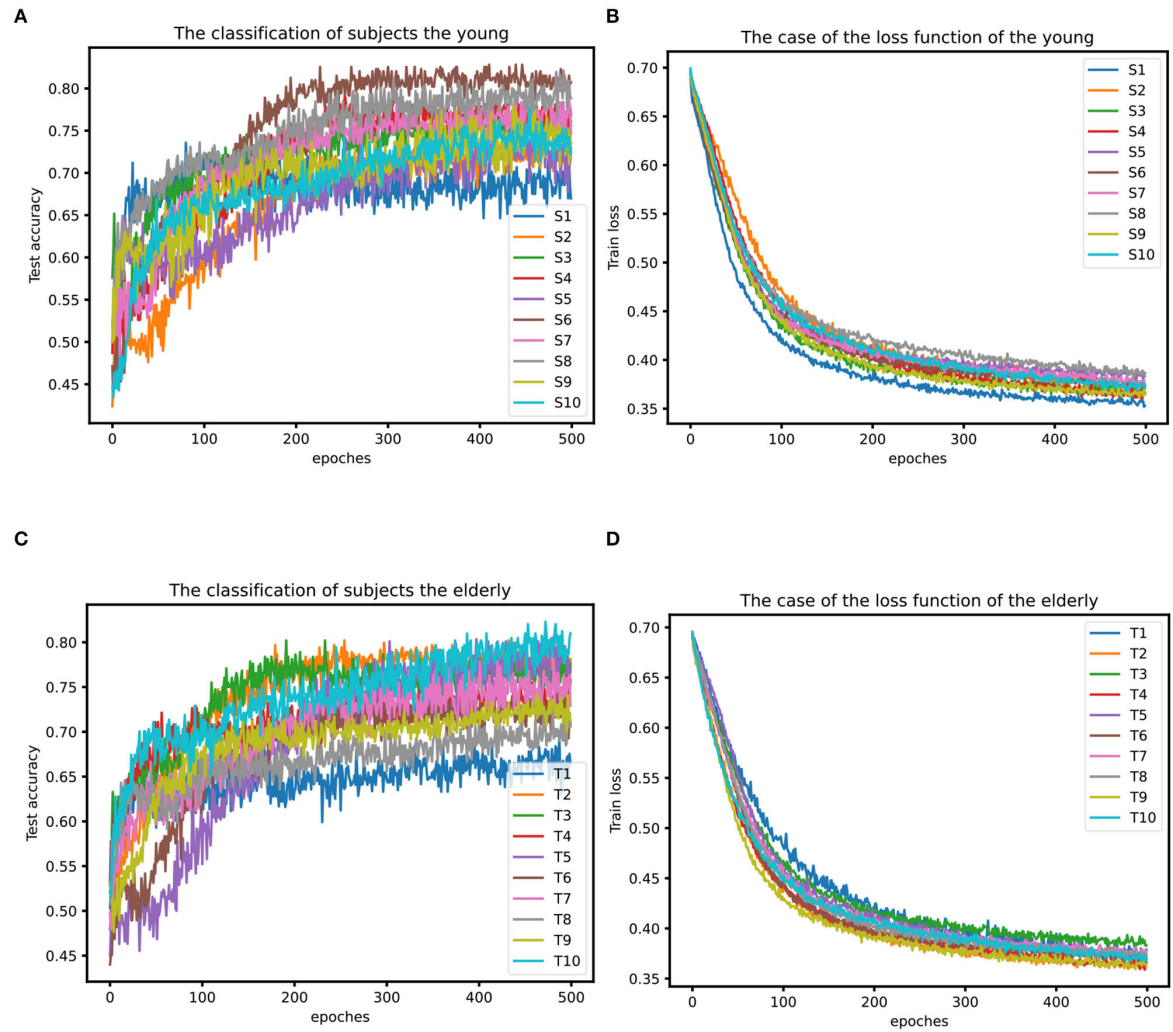
**(A)** Classification results of the young. **(B)** Loss function situation of the young. **(C)** Classification results of the elderly. **(D)** Loss function situation of the elderly.

For validating the classification method, the dataset was separated into 80% as the training set and the remaining 20% as the test set. At first, all the original samples of each subject were preprocessed, and then they were divided. At the same time, considering that the original sample size is too small and the neural network training model performance is insufficient, the sliding window method is introduced to expand the sample size of the data. The expansion steps are as follows: a 32 × 2,048 EEG fragment is obtained by taking the first 2 s as a time window, and a 32 × 512 sample is generated by taking the data with the 4-point interval from the fist point. The starting position moves to the second, the third, and the fourth point for further sampling. In this way, a 2 s time window is divided into four samples. Meanwhile, to avoid a high repetition rate between samples, the next 2 s time window started from 1 s, and the following 2 s time window began on 2 s. With the data enhancement, each original sample was converted into 12 32 × 512 samples, and the total data dimension of the experiment became 1,920 × 32 × 512. Then, it divided the training set and test set according to the ratio of 8:2 mentioned above, and the training set with about 1,536 samples and the test set with about 384 samples would be produced (except for S8), and compared the elderly and young people separately, as shown in Figure 11.

From Figures 11A,B, it can be seen that the global average accuracy rate of young people is higher than that of the elderly group, with a peak value of 82.81%, while the average and maximum accuracy rates of the elderly are lower than those of young people. In addition, the overall accuracy rate for most of the young subjects was above 0.7, while the older ones were a little lower, around 0.7. During the training stage, the epochs of young subjects gradually tended to stabilize around 240 iterations, while most older ones stabilized after 350 iterations, and the data quality of young persons is slightly better than that of the elderly. In addition, from Figures 11B,D, it also can be seen that the difference in the loss function of different young people is significantly larger than that of different elderly people, and the difference in data quality between young persons may be slightly larger than that between elderly individuals.

**Figure 12:**
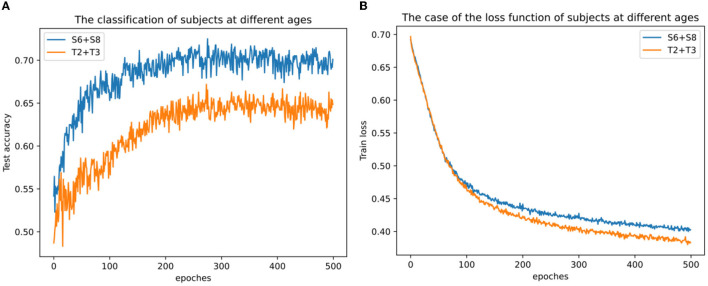
Classification results across subjects with mixed datasets. **(A)** Classification results across subjects with mixed datasets at different ages and **(B)** loss function across subjects with mixed datasets at different ages.

In addition, it also attempted classification tests on the cross-sample dataset for each age group. Here the data of two subjects S6 and S8 were selected as the mixed dataset of young adults, and the data of two subjects of T2 and T3 as the mixed dataset of old ones. It can be clearly seen from Figure 12A that the average accuracy of the youth mixed sample set is higher than the two data sets of the elderly mixed sample, and the highest accuracy rate of the mixed sample of young people can reach 0.725, indicating that the quality of mixed data between young individuals is still better than that of older individuals. The loss function on the training set is different. From Figure 12B, it can be seen that the decline rate of the mixed sample of young adults is lower than that of the mixed sample data of the elderly, probably because the mixed sample of S6 and S8 has 80 more trials than that of T2 and T3, and this result is also consistent with the highest loss function for S8 in Figure 11B. At the same time, due to the differences among subjects, the classification accuracy on the mixed dataset is lower than the classification accuracy on each separate dataset.

## Discussion

The analysis of the trend of decreasing ERD significance with the increase of experimental time is conducted, and the average of the RE of the six channels on the prefrontal lobe region is calculated. From the function activity aspect, the work on synchronization between the signal amplitudes and the phase has been discussed (Rosenblum et al., 1996; Nolte et al., 2004). It hypothesizes that integrating these complementary features will allow a better characterization of the BCI-related mental states and that including them in the feature extraction block, which is helpful to increase the BCI accuracy compared to standard approaches solely based on power spectra (Cattai et al., 2019). In this way, the phase relationship between EEG signals on the prefrontal and parietal lobes will be characterized by PLV. Aiming at the calculation and analysis of classification accuracy of 10 young subjects and 10 older ones in the previous sections, it analyzed the correlation between classification accuracy and the PLV, which is between the fatigue-sensitive channels on the parietal lobe and the specified channels in the frontal lobe for RE calculation.

Combined with the above conclusions, the average fatigue value of 10 young subjects was 32.086, and that of 10 older subjects is 25.796. The average fatigue value of young persons is higher than that of the elderly, indicating that young adults are more prone to fatigue during MI, which might be caused by their more concentrated attention during the MI. At the same time, the results of the PLV obtained above are statistically analyzed, with the channels whose PLV is significantly higher than the mean value of the corresponding test group removed. The average PLV of the young is 0.398, and that of the elderly is 0.270. The average PLV of young persons is higher than that of the elderly, indicating that compared with the elderly, the parietal lobe fatigue sensitive channels and specified prefrontal lobe channels have higher synchronization. From the perspective of fatigue, RE, and PLV, young persons are more likely to concentrate on MI tasks and thus fatigue is caused. The elderly subjects make more cognitive efforts to complete the task, but the MI classification accuracy is slightly lower than that of young adults, and it could hereby be inferred that age may affect the ability of MI.

According to the classification accuracy results mentioned above, the correlation between the PLV of young subjects and the elderly subjects to each classification accuracy is calculated here. For 10 young subjects, the correlation coefficient between the average PLV from the fatigue-sensitive channel P6 and the classification accuracy was 0.760, while for 10 elderly subjects, the correlation coefficient between the average PLV from the fatigue sensitive channel P2 and the classification accuracy was 0.731. The results show that there is a strong positive correlation between the classification accuracy and the average PLV of the fatigue-sensitive channel to the specified prefrontal lobe channel in both young and older subjects.

As a deep learning method, a CNN model is established for detecting left and right hands MI using a Muse headband that has potential use on older adults, but the experiment is performed on four healthy users aging from 33 to 55 years (Garcia-Moreno et al., 2020). More than 90% accuracy is achieved, however, the classification method beyond the EEG headset still requires further evaluation on aging people. For cognitive tasks, it indicates that robust plasticity of the prefrontal cognitive control system was found in the aging brain, and it also provides a custom-designed video game environment to assess cognitive abilities across the lifespan of human subjects (Anguera et al., 2013). Therefore, it is necessary to apply a CNN method by introducing the EEG data from channels on both frontal and parietal lobes for the MI-EEG classification by considering the ERD and fatigue phenomenon together in the aging group.

It also mentions that change in the location of activation in the brain is be more bilateral throughout the aging process. In that case, the application of sensorimotor rhythm (SMR) for BCI based on spatial information such as common spatial pattern (CSP) and Laplacian filtering becomes not so effective (Bashashati et al., 2007). The study also showed that age-related electrophysiological changes in healthy older adults significantly affected SMR characteristics in EEG, and found that the classification accuracy in the elderly is significantly lower than in the younger population by 15.9% (Chen et al., 2019). It means that the traditional classification methods depend more on the input features from the CSP enhancement. Compared with this pipeline mode, as an end-to-end method, CNN does not require these intermediate results of feature extraction, which can make full use of the features from the EEG signals. Therefore, the CNN classification accuracy keeps around 70% on the 10 older subjects by selecting 32 channels in the frontal and parietal regions, which have a close performance to young subjects. However, the number of young and older subjects is limited in the current study, and all the subjects are healthy ones. The classification strategies of the MI-BCI system for those who suffer neurological diseases such as stroke rehabilitation will be investigated in the future.

## Conclusion

The primary finding of our study is that the elderly are less affected by the level of fatigue during MI, even though the MI energy of the elderly is lower than that of the young. However, the deep learning method by extracting frontal and parietal channel data can be still suitable for the elderly, and the classification accuracy on MI tasks is at the acceptable levels of around 70% by CNN. It could be inferred that future BCI for the elderly population on MI will not merely depend on the SMR, and appropriate algorithms can be applied without obvious lateralization of ERD. However, the CNN model based on fused spatial information greatly improves the accuracy of the classification and leads to a longer training time. Supported by the rehabilitation robot previously developed, more participants will attend the EEG data collection, and an improved CNN classification method for real-time BCI based on MI would be attempted in the future.

## Data Availability Statement

The raw data supporting the conclusions of this article will be made available by the authors, without undue reservation.

## Ethics Statement

The studies involving human participants were reviewed and approved by Ethics Committee of West China Hospital of Sichuan University. The patients/participants provided their written informed consent to participate in this study.

## Author Contributions

XL and PC wrote the manuscript. NJ provided the suggestion of experiment design. PC and XY analyzed the EEG data and implemented the CNN classification method. All authors contributed to the article and approved the submitted version.

## Funding

The work has been financially supported by the National Natural Science Foundation of China (Grant Nos. 51805449 and 62103291), the Sichuan Science and Technology Program (Grant Nos. 2021ZHYZ0019 and 2022YFS0021), and the 1 · 3 · 5 project for disciplines of excellence, West China Hospital, Sichuan University (Grant Nos. ZYYC21004 and ZYJC21081). All findings and results presented in this paper are those of the authors and do not represent the funding agencies.

## Conflict of Interest

The authors declare that the research was conducted in the absence of any commercial or financial relationships that could be construed as a potential conflict of interest.

## Publisher's Note

All claims expressed in this article are solely those of the authors and do not necessarily represent those of their affiliated organizations, or those of the publisher, the editors and the reviewers. Any product that may be evaluated in this article, or claim that may be made by its manufacturer, is not guaranteed or endorsed by the publisher.
